# Prevalence and Morbidity Data on *Schistosoma mansoni* Infection in Two Rural Areas of Jequitinhonha and Rio Doce Valleys in Minas Gerais, Brazil

**DOI:** 10.5402/2013/715195

**Published:** 2013-03-19

**Authors:** Maria José Conceição, Aline Eduardo Carlôto, Eric Vinaud de Melo, Iran Mendonça da Silva, José Rodrigues Coura

**Affiliations:** ^1^Department of Preventive Medicine, Infectious and Parasitic Diseases Post-Graduation, Clementino Fraga Filho Hospital, Universidade Federal do Rio de Janeiro (UFRJ), 21045-900 Rio de Janeiro, RJ, Brazil; ^2^Laboratory of Parasitic Diseases, Instituto Oswaldo Cruz, Fiocruz, Manguinhos, 21045-900 Rio de Janeiro, RJ, Brazil; ^3^Tropical Medicine Post-Graduation, Fiocruz, 21045-900 Rio de Janeiro, RJ, Brazil; ^4^University of the State of Amazonas (UEA), 69077-000, Manaus, AM, Brazil

## Abstract

*Objective*. This study aimed to compare the prevalence and morbidity data on *Schistosoma mansoni* infection in two rural areas: the Jequitinhonha valley (area 1) and the Rio Doce valley (area 2) in the state of Minas Gerais, Brazil, covering the period from 2007 to 2010. *Material and Methods*. The parasitological stool tests were based on the quantitative method of Kato modified by Katz et al. Three clinical forms were considered: type I—schistosomiasis infection, type II—hepatointestinal form, and type III—hepatosplenic form. *Results*. The prevalence of infection among inhabitants of area 1 was 22.9%, with 2.1% presenting the hepatosplenic form and two cases of schistosomal myeloradiculopathy. The infection prevalence rate in area 2 was 20.2%, with 3.3% presenting the hepatosplenic form. *Conclusion and Recommendation*. There was no difference in the prevalence and in the morbidity of *Schistosoma mansoni* infection between the two areas, but it was predominant in young men with a low intensity of infection. The cases of schistosomal myeloradiculopathy in area 1 can be highlighted: these emphasize that schistosomiasis should not be neglected in Brazil. The lack of infection control in both areas may be related to the poor sanitation system, the absence of previous treatment, and the reinfection process.

## 1. Background

Surveys on infection by *Schistosoma mansoni* that have been conducted in Minas Gerais since the 1970 decade have aimed to follow up the natural history of schistosomiasis with emphasis on (1) the pathogenicity of the etiological agent and its experimental behavior [1], (2) the host's characteristics and the influence of race, occupation, education level, age group, sex, specific treatment and frequency of contact with water streams for the activities of washing clothes, leisure, and fishing [2, 3], and (3) environmental conditions, distribution of streams and their distance to homes, presence of *Biomphalaria*, and its infection rates and infectivity, with comparison of exposure to different isolates of the parasite [4, 5]. The morbidity of infection has been evaluated through clinical and epidemiological studies in two field areas, in the Rio Doce and Jequitinhonha valleys of the state of Minas Gerais, Brazil. Patients with hepatosplenic forms have been evaluated at the University Hospital of the Federal University of Rio de Janeiro, Brazil. The aim of the present work was to compare the prevalence of infection by *S. mansoni* in these two areas, the morbidity due to schistosomiasis according to classification of clinical forms of the disease.

## 2. Study Areas

The present survey was carried out in two rural areas of Minas Gerais: area 1—São João, and area 2—municipality of Capitão Andrade, located, respectively, in the Jequitinhonha and Rio Doce valleys, at distances of 890 km and 600 km from the state of Rio de Janeiro. The total number of inhabitants taking part in the survey was evaluated according to a random number table [6]. In area 1, 75 homes were sampled, involving 288 individuals; and in area 2, 80 homes with 257 individuals.

## 3. Methodology

### 3.1. Prevalence of *S. mansoni*

Stool parasitological tests were based on the Kato method, as modified by Katz et al. [7], and the intensity of infection was evaluated using the median number of eggs per gram of feces, according to the inhabitants' ages and gender.

### 3.2. Clinical Examination

In order to measure liver and spleen sizes, the subjects were examined in the dorsal position (during deep expiration) and in the Schuster position. Ultrasonography was requested to determine the portal vein caliber, liver and spleen sizes, and fibrosis. This study was approved by the Ethical Committee of the Instituto de Pesquisas Clínicas Evandro Chagas-Fiocruz (CONEP Protocol 0127.0.011.009-05).

The clinical classifications on both populations were based thus in three clinical forms: type I—schistosomiasis infection, with or without symptoms, which, if present, were moderate and not necessarily attributed to the disease; type II—hepatointestinal form, with frequent intestinal symptoms, such as dysenteric diarrhea and hepatomegaly; and type III—hepatosplenic form, with very frequent intestinal symptoms of dysentery and hepatosplenomegaly, with or without hematemesis or melena [8, 9].

During the clinical examinations on this population, the subjects were asked about intestinal symptoms of dysentery, hematemesis, and/or melena. Those with hepatosplenomegaly were admitted to the “Clementino Fraga Filho” University Hospital in Rio de Janeiro and were followed up by the teams of the Infectious and Parasitic Diseases Service and the Surgical Sector.

### 3.3. Study of Intermediate Host

Demarcated sites of water streams were examined for *Biomphalaria,* with the aims of classifying the mollusk species that were present and determining the cercarial infection rate, after exposure to light. This procedure was repeated over an eight-week period. If the *Biomphalaria* specimens were negative, they were infected experimentally with *S. mansoni* isolates from patients from different regions of Brazil, to test the mollusks' susceptibility to this infection.

### 3.4. Statistical Analysis

The statistical analysis was based on the chi-square test with a confidence level of 95% (*p* < 0.05).

## 4. Findings

### 4.1. Prevalence of Infection

A total of 288 parasitological stool tests were carried out on individuals in the Comunidade São João, comprising 163 females and 125 males. The prevalence rate of *S. mansoni* infection was 22.9% (33.6% in males and 14.3% in females). Men were twice as likely to acquire infection as women were (relative risk = 2.2), with the highest rate in the age group between 11 and 30 years old. There were 257 examinations on individuals in the population of Capitão Andrade (108 in males and 149 in females). The prevalence of infection was 20.2% (30.5% in males and 12.7% in females), predominantly in men (relative risk = 2.4) and in the age group between 11 and 30 years old. The number of *S. mansoni* eggs per gram of feces ranged from 24 to 48 in both areas, showing that the infection presented low intensity.

### 4.2. Clinical Forms of Schistosomiasis

In the São João community, 69.2% of the infected individuals presented the schistosomiasis infection form; 28.7% presented the hepatointestinal form; and 2.1% presented the hepatosplenic form. Two cases (one is 12 years old and the other is 13 years old) had the diagnosis of schistosomal myeloradiculopathy, for which the main symptoms were headache, limb and back pain, weakness, paresthesia, and paraplegia. These two patients underwent neurological examination and the venereal disease research laboratory, HIV antibody, cytomegalovirus, and herpes virus tests. These laboratory results were negative. Magnetic resonance imaging revealed dilatation of the medullary conus, and the likely source was indicated as schistosomal myelopathy. After treatment with a single oral dose of praziquantel (40 mg/kg), the symptoms regressed, with no sequelae over a three-year follow-up period.

In Capitão Andrade, 70.2% of a total of 65 positive inhabitants of the studied sample were classified as presenting the schistosomiasis infection form; 26.5% presenting the hepatointestinal form; and 3.3% presenting the hepatosplenic form (Table 1). The presence of esophageal varices was not confirmed through digestive endoscopy in the cases of the individuals with hepatosplenomegaly. No cases of ascites, cirrhosis, or other complications indicating liver involvement caused by comorbidity associated to *S. mansoni* infection was observed.

### 4.3. Intermediate Hosts

Mollusks from the genus *Biomphalaria glabrata* were the intermediate hosts in both areas. None of them were found to be positive for cercarial elimination during the period of the study, but they were seen to be susceptible when infected by *S. mansoni* isolates of patients from different regions of Brazil. The mechanism of infection of those people should be probably related to cercarial dispersion [10].

## 5. Discussion

Current studies have already described morbidity and prevalence results relating to *Schistosoma mansoni* worldwide. This disease affects at least 240 million people around the world, and more than 700 million people live in endemic areas [11]. Moreover, the infection is prominent in nineteen Brazilian states, especially in Minas Gerais, which accounts for about 70% of the endemic areas for the disease [12, 13]. The longitudinal surveys started in Minas Gerais emphasized that the morbidity and prevalence rates decrease when a specific treatment of infected individuals is associated with improvements of sanitary conditions [14]. Different authors in Brazil have contributed towards studies on the influence of treatment on prevention of severe clinical forms of this disease [15–19].

There was no difference in the prevalence of infection by *S. mansoni* between São João (22.9%) and Capitão Andrade (20.2%). Both areas highlighted a gradual increase in the rate up to the age of 11 years, with a decrease after the age of 50 years. The prevalence of rates higher than 20.0% in both areas indicates that these areas are included in category II with moderate prevalence (≥20% and <50%) [20]. There was higher prevalence among males than that among females, such that men were twice as likely to become infected as women were. The intensity of infection was low, ranging from 24 to 48 eggs per gram of feces [21].

In both areas, the sewage systems were poor. Regarding water supply, it was more precarious and poor in quality in São João, where the current study began. No comparisons can be made with any previous surveys in this area, although two cases of schistosomal myeloradiculopathy can be highlighted. These showed regression of symptoms, without sequelae, after treatment with praziquantel and monitoring of case evolution for three years after the infection had been treated. Similar clinical forms were presented for Brazilian authors [22, 23].

## 6. Conclusion

There was no difference in the prevalence of infection by *S. mansoni* between São João and Capitão Andrade, with a low intensity of infection and a higher rate among males up to the age of 11 years. It was highlighted two cases of myeloradiculopathy in the Jequitinhonha valley, with regression of symptoms after specific treatment.

There was not infection control in both areas. The factors determining this lack of control may include low sensitivity and the small number of diagnostic procedures performed, as well as no response to the drugs used.

Other additional factors that might explain the maintenance of transmission rates include the poor sanitation system, the lack of early specific treatment, and the reinfection processes.

## Figures and Tables

**Table 1:** Prevalence rates and clinical forms of Schistosomiasis mansoni in Comunidade São João (area 1) and Capitão Andrade (area 2) in Minas Gerais.

Prevalence/clinical forms	Comunidade São João			Capitão Andrade		
	*N*	*P*	%	*N*	*P*	%
Prevalence	288	65	22.9	257	78	20.2
Schistosomiasis infection	199	45	69.2	180	55	70.2
Hepatointestinal	83	18	28.7	68	21	26.5
Hepatosplenic form	6	2	2.1	9	2	3.3

*N*: total number of inhabitants, *P*: prevalence rates (*p* < 0.05).
